# Deletion of BST2 Cytoplasmic and Transmembrane N-Terminal Domains Results in SARS-CoV, SARS-CoV-2, and Influenza Virus Production Suppression in a Vero Cell Line

**DOI:** 10.3389/fmolb.2020.616798

**Published:** 2020-12-18

**Authors:** Alexander A. Dolskiy, Sergei A. Bodnev, Anastasia A. Nazarenko, Anastasia M. Smirnova, Olga G. Pyankova, Anna K. Matveeva, Irina V. Grishchenko, Tatiana V. Tregubchak, Oleg V. Pyankov, Alexander B. Ryzhikov, Elena V. Gavrilova, Rinat A. Maksyutov, Dmitry V. Yudkin

**Affiliations:** State Research Center of Virology and Biotechnology “Vector”, Rospotrebnadzor, World-Class Genomic Research Center for Biological Safety and Technological Independence, Federal Scientific and Technical Program on the Development of Genetic Technologies, Novosibirsk, Russia

**Keywords:** SARS-CoV, SARS-CoV-2, influenza, BST2, tetherin

## Abstract

SARS-CoV-2, which emerged in Wuhan (China), has become a great worldwide problem in 2020 and has led to more than 1,000,000 deaths worldwide. Many laboratories are searching for ways to fight this pandemic. We studied the action of the cellular antiviral protein tetherin, which is encoded by the BST2 gene. We deleted the transmembrane domain-encoding part of the gene in the Vero cell line. The transmembrane domain is a target for virus-antagonizing proteins. We showed a decrease in SARS-CoV-2 in cells with deleted transmembrane BST2 domains compared to the initial Vero cell line. Similar results were obtained for SARS-CoV and avian influenza virus. This finding may help the development of antiviral therapies competitively targeting the transmembrane domain of tetherin with viral-antagonizing proteins.

## Introduction

Bone marrow stromal cell antigen 2 (BST2), also known as tetherin, plays an important role in cellular antiviral response during infection by enveloped viruses mainly by preventing virus release as have been shown on human immunodeficiency virus 1 (HIV1) and Ebola virus (Gupta et al., 2009; Vande Burgt et al., 2015). However, tetherin antagonists encoded by certain viruses, such as HIV-1 Vpu protein and Ebola glycoprotein, have been shown to enhance the release of HIV-1 and Ebola viruses in fibroblasts and T cells treated by interferon-α (IFN-α) (Neil et al., 2008). In addition, previous studies showed BST2 inhibition by the spike (S) protein of SARS-CoV; and ORF7 of SARS-CoV encodes a protein that neutralizes BST2 by inhibiting its glycosylation (Wang et al., 2019). Finally, BST2 knockdown in Vero cell line increased the production of influenza A/H1N1 and A/H3N2 viruses as well as vaccinia virus and porcine epidemic diarrhea (PED) virus in a plaque assay (Yi et al., 2017).

The transmembrane tetherin protein is expressed in B-cells, bone marrow stromal cells, dendritic cells, and other cell types (Blasius et al., 2006; Erikson et al., 2011), and is located mainly in the cholesterol-rich domains (lipid rafts) of the plasma membrane (Neil et al., 2008; Yi et al., 2017). Tetherin protein contains four functional domains including cytoplasmic N-tail (CT), transmembrane domain (TM), extracellular domain (EC), and C-terminal domain (GPI). The EC domain is responsible for the direct binding of the released viruses (Arias et al., 2011; Hotter et al., 2013), while the TM domain have been shown to interact with the HIV-1 Vpu protein, thus antagonizing the virus production (Iwabu et al., 2009). Importantly, the impact of the tetherin protein domains on production of SARS-CoV and SARS-CoV-2 viruses remains unaddressed.

In this work, we generated a mutant Vero cell line containing a deletion within CT and TM domains encoding sequence of the BST2 gene using CRISPR/Cas9 system and investigated the production of SARS-CoV, SARS-CoV-2, and avian influenza viruses in these cells using quantitative real-time PCR approach.

## Materials and Methods

### Transgenic Cell Line Production

To generate a construct for CRISPR/Cas9, two RNA guides (gRNA1: GGAGTCGGGCCTGAAGTTAG, gRNA2: GACACTCCATCACTGCCCGG) have been sub-cloned into pSpCas9(BB)-2A-GFP plasmid (Addgene, #48138) using XbaI and PciI sites. The resulting plasmid was transfected into Vero cells (SRC VB “Vector” Rospotrebnadzor cell repository) with Lipofectamine 3000 (Thermo Fisher Scientific, USA) according to the manufacturer's protocol. The mutant clones of cells were identified by Sanger sequencing of PCR amplicons generated using the following primers: GGTCAGGACAGCTCCTATGCTA (forward) and AGATTATTGTCCTCCCTACCCC (reverse).

### Cells Infection With SARS-CoV and SARS-CoV-2

All experiments involving live SARS-CoV (Urbani, Erasmus University Medical Center, Rotterdam) and SARS-CoV-2 (nCoV/Victoria/1/2020) followed the approved standard operating procedures of the biosafety level-3 facility. The Vero cells were seeded into 24-well plates (100,000 cells per well) and infected with either SARS-CoV or SARS-CoV-2 at an MOI of 0.001 PFU/cell as described in Muth et al. (2018) and Hoffmann et al. (2020). In the experiment with trypsin, we added 4 μg/ml TPCK-treated trypsin (Sigma-Aldrich, USA). After 1 h of infection, the unbound viruses were removed by washing with Eagle's MEM serum-free medium. Next, 1 ml of 2 μg/ml TPCK-treated trypsin solution in DMEM/F12 (Biolot, Russia) have been added to the plates intended for cultivation in the presence of trypsin. The plates with subsequent cultivation without trypsin were supplemented with DMEM/F12 medium only. Followed by 48 h of cultivation at 37°C in 5% CO_2_, the total RNA was isolated by Riboprep kit (ILS, Russia) and the copy number of viral genomes was measured using SARS diagnostic kit (SRC VB “Vector” Rospotrebnadzor; Patent RU2733665C1), which utilizes TaqMan real-time PCR reaction with the following primers: 5′-GTTGCAACTGAGGGAGCCTTG-3′ (forward), 5′-GAGAAGAGGCTTGACTGCCG-3 (reverse) and 5′-FAM-TACACCAAAAGATCACATTGGCACCCG-BHQ1-3′ (probe). The plasmid pJet1.2_SARS containing a fragment of the SARS-CoV-2 MN997409.1 strain genome (positions 28670–28826) was used as a reference for qPCR normalization. The conversion of the viral nucleic acid concentration into the number of viral genome copies was performed by DNA Copy Number and Dilution Calculator (Thermo Fisher). Each experiment included three biological replicates. The statistical significance was calculated by a two-sample *t*-test. The differences were considered significant for *p*-values < 0.50.

### Cells Infection With Influenza Virus

All experiments with influenza viruses followed the approved standard operating procedures of the biosafety level-2 facility. The production of Avian influenza H5N1 (A/Nghe An/08VTC/, H5N1/Chany/) and H5N8 (A/Astrakhan/3111/2016) viral stocks, the infection of cell lines and hemagglutination inhibition (HAI) tests were performed as described previously (Nechaeva et al., 2019). In brief, viral stocks were produced in MDCK cells, and monolayers of Vero and Vero-BST2Δ221 cells was infected at an MOI of 0.01 PFU/cell in a 75 cm^2^ flask. For HAI, the assayed sera were pretreated with receptor destroying enzyme (RDE) (Denka, Japan). The hemagglutination reaction was performed in 96-well plates with 1% chicken red blood cells (RBCs). The HAI titer was determined as the reciprocal dilution of the last row, which contained non-agglutinated RBCs.

## Results

**Figure 1:**
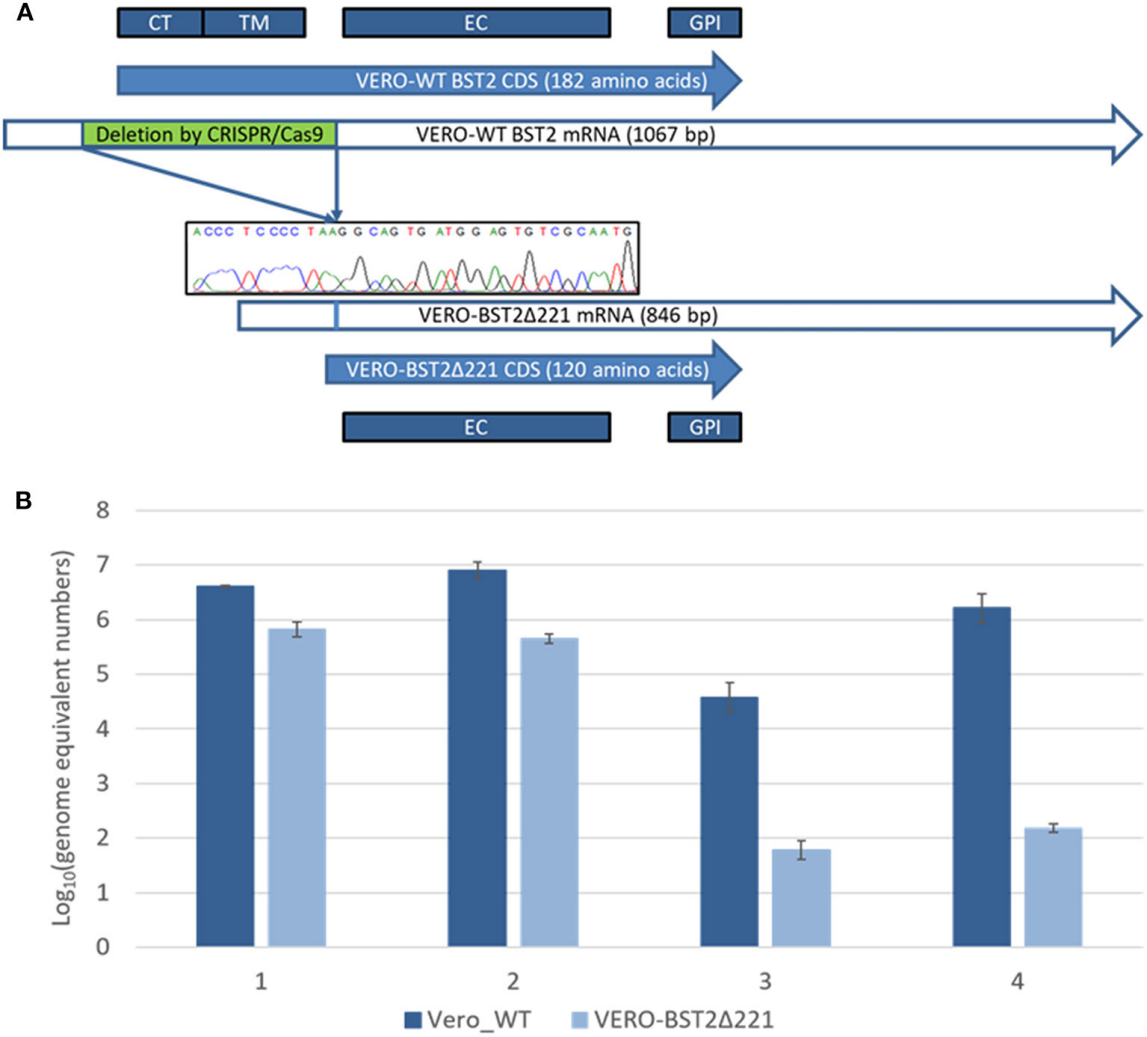
**(A)** Deletion of 221 nucleotides from the mRNA of the BST2 gene results in the loss of the CT and TM domains in the encoded protein. CT, cytoplasmic N-tail; TM, transmembrane domain; EC, extracellular domain; GPI, C-terminal domain. **(B)** Virus production represented as the following Log_10_ amounts of genome equivalent numbers: 1—SARS-CoV, trypsin; 2—SARS-CoV, without trypsin; 3—SARS-CoV-2, trypsin; 4—SARS-CoV-2, without trypsin.

Following the transfection of Vero cells with plasmid DNA encoding CRISPR/Cas9 effectors and the corresponding RNA guides, the partial homozygous deletion of BST2 gene was confirmed by Sanger sequencing in one of the clones obtained by single-cell dilution after lipofection and exhibited a loss of the 73–294 nucleotide region (ref. seq. XM_007995721.1). The absence of a frame shift was also confirmed by Sanger sequencing. The deleted part of the BST2 gene encodes the CT and TM domains (Figure 1A). The obtained Vero cell line was named Vero-BST2Δ221.

Next, we aimed to analyze the production of SARS-CoV (Urbani, Erasmus University Medical Center, Rotterdam) and SARS-CoV-2 (nCoV/Victoria/1/2020) in Vero-BST2Δ221 and control Vero cell lines. For that purpose, equal number of both cell types were infected with SARS-CoV or SARS-CoV-2 in the presence or absence of trypsin, and the number of viral genome equivalents was determined using real-time qPCR approach as described in MMs section. Remarkably, we observed about 10-fold decrease in SARS-CoV production in Vero-BST2Δ221 cells (Figure 1B) as compared to controls regardless of the infection conditions. For SARS-CoV-2, we demonstrated 30- and 40-fold decreases in virus production in the presence and absence of trypsin, respectively (Figure 1B).

Finally, to demonstrate the suppression of production of other enveloped viruses in Vero-BST2Δ221 cells, the latter were infected with avian influenza H5N1 (A/Nghe An/08VTC/, H5N1/Chany/) and H5N8 (A/Astrakhan/3111/2016) viruses. The subsequent hemagglutinating tests revealed 4-, 32-, and 8-fold decreases in viral titers in BST2Δ221 cultures as compared control cells, indicating a dramatic suppression of virus production in these cells (Table 1).

**Table 1:** Analysis of influenza virus production in Vero and Vero-BST2Δ221 cell lines by the hemagglutination inhibition (HAI) test.

**Influenza virus strains**	**Subtype**	**Hemagglutination inhibition (HAI) test**	
		**Vero**	**Vero-BST2Δ221**
A/Nghe An/08VTC/chicken	H5N1	256	64
H5N1/Chany/chicken	H5N1	64	≤ 2
A/Astrakhan/3111/2016/chicken	H5N8	16	≤ 2

## Discussion

Tetherin plays a key role in the cellular antiviral response by binding to released enveloped viruses, thus preventing subsequent infections. However, certain viruses produce antagonizing proteins to resist tetherin action, mainly by targeting the tetherin TM domain (Blasius et al., 2006; Neil et al., 2008; Wang et al., 2019). The exact mechanisms of interaction of viruses with BST2 protein through the TM domain and subsequent inhibition of viral production remain unclear, and the transmembrane location of the TM domain was only predicted (Roy et al., 2019). Additionally, protective five amino acid deletion in cytoplasmic domain of BST2/tetherin was found in human and ancient DNA of Neanderthal and Denisovan. This deletion prevents binding of BST2-antagonizing protein (Nef) of Simian immunodeficiency viruses and is supposed to pose a barrier for virus transmission from non-human primates to human (Sauter et al., 2011). Therefore, we hypothesized that the absence of a target motif for viral BST2-antagonizing proteins could lead to the inability of viruses to inhibit tetherin antiviral action. Consequently, we demonstrated that deletion of the sequence encoding CT and TM domains in the BST2 gene leads to a substantial impairment of coronavirus and avian influenza virus production in Vero cells. These findings may help to develop antiviral therapies, including anti-COVID-19 therapies, by targeting the CT and TM domains of tetherin gene *in vivo*. Such therapeutic agent can be antibody-based. In fact, anti-BST2 antibody was previously developed as a potent anticancer therapeutic. Based on observation that increased BST2 activity was accompanying endometrial cancer, anti-BST2 mAb has been developed and proved to exert a significant anticancer effect in experiments on mouse model (Hiramatsu et al., 2015). Specifically, based on above and on our results, we suggest that an agent capable of binding to the TM domain of the tetherin protein could potentially make it unavailable for viral BST2-antagonizing proteins action thus facilitating the cellular antiviral response in anti-enveloped viruses therapy.

## Data Availability Statement

The raw data supporting the conclusions of this article will be made available by the authors, without undue reservation, to any qualified researcher.

## Author Contributions

AD, SB, and DY conceived and designed the study. EG, RM, and DY supervised the study. AD and DY drafted the manuscript and analyzed the data. IG, OVP, AR, EG, and RM wrote, reviewed, and edited the manuscript. RM acquired the funding. AD, AM, IG, TT, and EG performed the molecular experiments. SB, AN, AS, OGP, OVP, and AR performed the virological experiments. AD created the figures. OVP, AR, and RM provided the resources. All authors read and approved the final manuscript.

## Conflict of Interest

The authors declare that the research was conducted in the absence of any commercial or financial relationships that could be construed as a potential conflict of interest.
